# Development of the Pneumococcal Genome Library, a core genome multilocus sequence typing scheme, and a taxonomic life identification number barcoding system to investigate and define pneumococcal population structure

**DOI:** 10.1099/mgen.0.001280

**Published:** 2024-08-13

**Authors:** Melissa J. Jansen van Rensburg, Duncan J. Berger, Iman Yassine, David Shaw, Andy Fohrmann, James E. Bray, Keith A. Jolley, Martin C. J. Maiden, Angela B. Brueggemann

**Affiliations:** 1 Nuffield Department of Population Health, University of Oxford, Oxford, UK; 2 Department of Biology, University of Oxford, Oxford, UK

**Keywords:** cgMLST, genotyping, genome library, population structure

## Abstract

Investigating the genomic epidemiology of major bacterial pathogens is integral to understanding transmission, evolution, colonization, disease, antimicrobial resistance and vaccine impact. Furthermore, the recent accumulation of large numbers of whole genome sequences for many bacterial species enhances the development of robust genome-wide typing schemes to define the overall bacterial population structure and lineages within it. Using the previously published data, we developed the Pneumococcal Genome Library (PGL), a curated dataset of 30 976 genomes and contextual data for carriage and disease pneumococci recovered between 1916 and 2018 in 82 countries. We leveraged the size and diversity of the PGL to develop a core genome multilocus sequence typing (cgMLST) scheme comprised of 1222 loci. Finally, using multilevel single-linkage clustering, we stratified pneumococci into hierarchical clusters based on allelic similarity thresholds and defined these with a taxonomic life identification number (LIN) barcoding system. The PGL, cgMLST scheme and LIN barcodes represent a high-quality genomic resource and fine-scale clustering approaches for the analysis of pneumococcal populations, which support the genomic epidemiology and surveillance of this leading global pathogen.

**Impact Statement**

Many thousands of pneumococcal genomes are publicly available, and this creates opportunities for the scientific community to re-use existing data; however, these data are most useful when the contextual data (provenance and phenotype) are also linked to the genomes. Therefore, we created a curated, open access database in PubMLST that contained nearly 31 000 published pneumococcal genomes and the corresponding contextual data for each genome. This large and diverse pneumococcal database was used to create a novel cgMLST scheme and multilevel clustering method to define genetic lineages with high resolution and a standardized nomenclature. These are open access resources for all to use and provide a stable framework for the characterization of global pneumococcal populations.

## Data Summary

The PGL, cgMLST scheme, and LIN barcodes are available from PubMLST (https://pubmlst.org/bigsdb?db=pubmlst_spneumoniae_isolates_pgl). Genomes are available in PubMLST via ID numbers and accession numbers listed in Data S1. The code used for data analyses is available at https://github.com/brueggemann-lab/pgl_cgmlst_2024. The authors confirm all supporting data, code and protocols have been provided within the article or through supplementary data files.

## Introduction

In the first two decades of the twenty-first century, the capacity to sequence the whole genome of microbes transformed the fields of microbiology and infectious disease. Furthermore, genomic epidemiology and surveillance are playing increasingly important roles in public health, vaccine development and the assessment of vaccine impact [1,4]. Thousands of bacterial genome sequences are publicly available, and the potential for re-use of these genomes is of major benefit to the scientific community.

*Streptococcus pneumoniae* (the pneumococcus) is a bacterium that primarily resides in the healthy human nasopharynx but is also a major cause of localized and invasive infections worldwide. In 2019, prior to the COVID-19 (coronavirus disease 2019) pandemic, pneumococci were estimated to cause over 650 000 (95 % uncertainty interval (UI) 553 000–777 000) deaths due to pneumonia and nearly 45 000 (95 % UI 34 700–59 800) deaths from meningitis among people of all ages [5]. Non-pharmaceutical interventions implemented during the pandemic to contain the spread of SARS-CoV-2 led to significant and sustained reductions in invasive pneumococcal disease (IPD), but IPD is returning to pre-pandemic levels in many countries [6,7]. Pneumococcal conjugate vaccines are used in many countries worldwide and have successfully reduced the global burden of pneumococcal disease over the past 20 years; however, the pandemic disrupted vaccination programmes worldwide, and restoring these programmes is a public health priority [8].

As of 2023, there were tens of thousands of pneumococcal genome sequences in public repositories including the European Nucleotide Archive (ENA) and the National Center for Biotechnology Information (NCBI); however, the ENA and NCBI repositories often include minimal corresponding contextual data (provenance and laboratory data), and often the genomes are only available as unassembled short-read data, which limits their use by many investigators. Therefore, the first objective of this study was to create a Pneumococcal Genome Library (PGL) compiled with assembled genomes and corresponding contextual data from the peer-reviewed published literature and make those data freely available to all users via the PubMLST platform. We also incorporated basic genome quality criteria into the PGL to enable users, including non-specialists, to query, filter, re-analyse and download published pneumococcal genomes.

Originally, the pneumococcal population structure was defined using seven-locus MLST and clonal complexes (CCs; clusters of related isolates based on MLST loci), but recently, whole genome sequences have been used to define genetic lineages more precisely [9,12]. This has led to the development of different genotyping and clustering methods for pneumococci based on whole genome sequence data, including Global Pneumococcal Sequence Clusters (GPSCs) and Mandrake clustering [11,12]. A well-defined population structure is the foundation on which other criteria can be mapped and interpreted, such as the lineages causing disease versus those found among healthy individuals, or antimicrobial-resistant lineages. Understanding the expected pneumococcal population structure is also necessary to detect and interpret any observed population-level changes to that structure. For example, pneumococci typically possess a polysaccharide capsule (serotype), and this is the primary vaccine antigen. There are over 100 antigenically different serotypes, and these are typically associated with specific pneumococcal genotypes; therefore, new serotype/genotype combinations are a potential indication of capsular switching events [13,15].

The MLST approach of defining alleles based on sequence variation at a defined set of loci was transformative because sequence data are unambiguous and easily portable, and a common nomenclature was defined [16,17]. Assigning alleles and STs from large numbers of genomes is easily automated, while still relying on the expertise of a human data curator to ensure high-quality data. Genomes can also be assessed using ribosomal MLST (rMLST), which characterizes allelic diversity at the 53 bacterial ribosomal genes and is especially useful for species identification [18,19].

Core genome MLST (cgMLST) schemes have been implemented for several global pathogens to assess sequence variation at hundreds of core genes across the bacterial genome and increase the resolution of defined genetic lineages [20,25]. Therefore, the second objective of this study was to develop and implement a cgMLST scheme for pneumococci, which we extended to include a taxonomic life identification number (LIN) system to improve the resolution and clustering of genetic sublineages of pneumococci. The LIN approach was originally implemented for *Klebsiella pneumoniae* and has the main advantage of providing a definitive and stable ‘barcode’ for each genome that can be used to define and cluster groups of genetically related isolates, based on sequence variation within the core genes used in the cgMLST scheme [26,27].

Here, we describe the development of the PGL as a community resource of nearly 31 000 assembled pneumococcal genomes with their corresponding contextual data. We used the PGL to design a robust and reproducible pneumococcal cgMLST scheme of 1222 core genes and developed a LIN barcoding system to cluster pneumococci across multiple levels, based on sequence variation at those 1222 genes. We implemented the PGL, cgMLST scheme, and LIN codes within PubMLST to provide open access resources for the scientific community and a standard nomenclature for the characterization of global pneumococcal populations.

## Methods

### Literature search and article eligibility

We searched PubMed on 31 January 2020 using the terms ‘(“*Streptococcus pneumoniae*” OR “pneumococcus”) AND (“genome sequencing” OR “genome sequence” OR “Genome, Bacterial”)’. An additional search was carried out on 15 July 2020 using the terms ‘(“*Streptococcus pneumoniae*” OR “pneumococcus”) AND “genome sequencing”’. Titles and abstracts of full-text, peer-reviewed articles published in English between 1 January 2000 and 15 July 2020 were manually screened for eligibility, and methods and results sections of an article were also reviewed if necessary. Eligible publications were original research articles that described genome data from at least one naturally occurring pneumococcal isolate.

### Genome data availability

The text and supplementary files of eligible published articles were searched for International Nucleotide Sequence Database Collaboration (INSDC) accession numbers, identifiers from other public databases or genome data. For each article, we confirmed that the number of accessions, identifiers or data files provided matched the total number of genomes described in the text, including any reference genomes and supplementary genome data. We also confirmed that any INSDC accession numbers were valid and corresponded to pneumococcal records.

### Data acquisition and genome assembly

Contextual data (country of origin, year of isolation, source, diagnosis, sex, age and serotype) were extracted from the published article and supplementary files, and data were manually cleaned to conform to allowed values accepted by the *S. pneumoniae* PubMLST database. If available, assembled genomes were downloaded from the NCBI Nucleotide/Assembly databases. When assemblies were not available, short-read data was downloaded from ENA, and genomes were assembled *de novo* using Velvet (v1.2.10) and VelvetOptimiser (v2.2.4) using a range of kmer sizes (19–191 bp) [28]. Assembled contigs shorter than 200 bp were removed. Genomes generated in earlier studies were assembled as previously described [9,10, 29,40]. Illumina MiSeq data from two datasets were assembled poorly with Velvet, so these genomes were assembled with SPAdes implemented in the INNUca pipeline v.4 [41,44]. Velvet assemblies were retained for the subset that did not assemble with the INNUca pipeline.

### Creation of the PubMLST Pneumococcal Genome Library

In the *S. pneumoniae* PubMLST database, isolate records were created that included the assembled genome, any corresponding provenance or laboratory data and the PubMed identifier of the original publication. A separate view of the *S. pneumoniae* PubMLST database was created to enable users to access the PGL directly (https://pubmlst.org/organisms/streptococcus-pneumoniae/pgl). All known MLST and rMLST alleles were assigned by the BIGSdb autotagger tool in PubMLST [19]. New alleles and STs were manually curated and assigned by the database curators (ABB and JEB, respectively).

### rMLST species identification

The rMLST database (https://pubmlst.org/species-id) contains bacterial genomes compiled from the NCBI Assembly database and the assembly of short-read data [18,19]. The allelic variants of the rMLST genes of these genomes have been fully catalogued. For species identification purposes, the lowest common taxonomic node (LCTN) of each rMLST allele is calculated based on the species annotations of the genomes that have that allele. For example, an allele observed in multiple *Streptococcus* species is assigned an LCTN of *Streptococcus* (a genus node), whereas an allele only observed in pneumococcal genomes is assigned an LCTN of *S. pneumoniae*.

The rMLST species identification process includes three stages. First, the query genome is scanned against the rMLST allele library using blastn (v. 2.12.0), and exact allelic matches are recorded [45]. Second, the LCTNs of the matched alleles are mapped onto the nodes of the bacterial taxonomic hierarchy (as defined by NCBI Taxonomy). This involves incrementing a counter for each node in the hierarchical path from the phylum node to the LCTN. For example, an allele with an LCTN of *Streptococcus* will increment the nodes: Bacillota (phylum), Bacilli (class), Lactobacillales (order), Streptococcaceae (family) and *Streptococcus* (genus). This process is repeated for all the derived LCTNs, and only the lowest positive-count nodes within each branch of the taxonomic hierarchy are reported in the results. Finally, the 'allele support' of each reported taxonomic node is calculated based on the number of rMLST alleles found within the query genome. Allele support is defined as the number of query genome alleles observed for the reported node divided by the total number of query genome alleles observed across all reported nodes (expressed as a percentage). A reported species node with an allele support above 90% indicates a high degree of confidence in that result.

### Genome quality control

The quality of each genome sequence was assessed based on several criteria. First, rMLST species identification was applied to each genome and interpreted as pass (only *S*. *pneumoniae* detected, support ≥90%), warning (only *S pneumoniae* detected, support <90%) or fail (*S. pneumoniae* not detected or *S. pneumoniae* plus other organisms detected). Second, genomes were evaluated for evidence of mixed MLST and rMLST alleles: the presence of >1 allele at any MLST or rMLST locus (excluding putatively paralogous genes BACT000014 [*rpsN*, 30S ribosomal protein S14] and BACT000062 [*rpmG*, 50S ribosomal protein L33]; https://pubmlst.org/species-id) flagged genomes that were potentially contaminated with non-pneumococcal DNA or consisted of multiple pneumococcal strains.

Third, the interquartile deviation method was used to develop data-derived thresholds for genome size, GC content, number of contigs, *N*_50_, number of Ns and number of gaps. Minimum and maximum thresholds (*T*) were set using the following equations, using *c* = 1.5 for ‘soft’ thresholds and *c* = 2.2 for ‘hard’ thresholds:

$$T_{min}=Q1-(c*IQR)$$

$$T_{max}=Q3+(c*IQR)$$

For each metric, genomes were categorized as ‘pass’ (between the soft thresholds), ‘warning’ (between the hard and soft thresholds) or ‘fail’ (outside the hard thresholds). The results of the rMLST species identification, MLST/rMLST allele curation, and genome quality thresholds were implemented in the *S. pneumoniae* PubMLST database to enable users to filter PGL data based on genome quality. Finally, genome completeness was assessed using BUSCO (Benchmarking Universal Single-Copy Orthologs) (v.5.4.3) and the lactobacillales_odb10 lineage dataset [46].

### Definition of a cgMLST genotyping scheme

Seventy-one complete (closed) pneumococcal genomes were available for download from NCBI in March 2019, of which 29 were excluded from analyses (non-RefSeq genomes, *n* = 8; genomes with gaps, *n* = 5; and genomes of STs represented more than once, *n* = 16, i.e. only one representative of each ST was selected). The chewBBACA software suite (v2.0.16) was run on the remaining 42 reference (RefSeq) genomes using default parameters to create and validate a cgMLST scheme [47]. CreateSchema identified 3139 complete, non-redundant coding sequences in each genome, and alleles were defined for each of these genes using AlleleCall. Putatively paralogous genes (*n* = 44) were removed using RemoveGenes, and annotations for each gene were retrieved using UniprotFinder. Among the remaining 3095 genes, 1385 (44.8 %) were detected in >95% of reference genomes, excluding genes with alleles that were 20% larger or smaller than the modal length of the distribution of matched genes.

### cgMLST scheme validation and refinement

The cgMLST scheme was assessed using 8263 genomes from an early subset of the PGL, of which 86 were excluded from further analyses: likely contaminated based on the presence of multiple alleles at rMLST loci (*n* = 74), overall size ±2 standard deviations from the mean pneumococcal genome size (*n* = 11), not a pneumococcus, based on rMLST species identification or seven novel MLST alleles (*n* = 1). The chewBBACA AlleleCall command was re-run on the 8177 genomes using the 1385 gene scheme. A further 629 genomes were removed due to high numbers of missing genes (>33) and or warnings (>26). One duplicated genome (two genomes linked to the same ENA accession number) was removed. The remaining 7547 genomes comprised the cgMLST scheme validation dataset. AlleleCall flagged 242 genes for manual review, and 25 of these were removed from the scheme for these reasons: frequent absence (*n* = 15), alleles 20 % smaller than the length mode of the distribution (*n* = 6), transposases (*n* = 2), paralogous hits (*n* = 1) or frequently at the end of assembly contigs (*n* = 1). Finally, a provisional 1360 gene cgMLST scheme was implemented in PubMLST.

Initial seed alleles were identified using the validation set of 8177 genomes plus an additional 796 recently assembled genomes. chewBBACA AlleleCall was used to identify alleles, and only those at the mode length for each gene were retained. Automated curation within PubMLST (using the BIGSdb scannew.pl script and thresholds of 98% sequence identity and 98% alignment length to existing alleles) was performed on the 7547 genomes in the validation dataset. In brief, alleles were assigned by nucleotide sequence comparison to all known alleles at that locus. Sequences that exactly matched known alleles were automatically assigned the allele by the autotagger in BIGSdb (autotag.pl) that ran on new data. Putatively new alleles were flagged and then reviewed and assigned by a human curator. For genomes where alleles/genes were not identified, additional blastn searches were performed using lower thresholds of ≥70 % sequence identity and at least 50% gene length. Truncated alleles and gene sequences containing insertion sequences, mobile genetic elements or that were otherwise interrupted, were not assigned allele numbers. Manual (human) inspection and curation of 216 genes were performed to define any alleles not assigned automatically, using blastn with a threshold of 95 % sequence identity to any known allele. There were 138 core genes that contained a high proportion of disrupted or missing alleles and were thus excluded since they would be poorly reproducible in a typing scheme. Ultimately, 1222 core genes were included in the final cgMLST scheme, and a core genome sequence type (cgST) was assigned for all PGL genomes that had allele assignments at >97% of core genes (i.e. maximum 25 missing alleles). EggNOG-mapper (v5.0.0) was used for functional annotation of cgMLST genes using one randomly selected representative of each coding sequence [48]. PhiPack software was used to conduct an intragenic recombination analysis using the pairwise homoplasy index method with default parameters [49].

### Core genome sequence type and LIN code assignment

A LIN code is a multi-position, integer-based code where each position (‘bin’) corresponds to a cgMLST allelic mismatch value [50]. The pneumococcal LIN code system consists of 11 bins. The first five bins represent the deepest hierarchical levels of relatedness: species (1180 allelic mismatches), superlineage (750 allelic mismatches), lineage (540 allelic mismatches), sublineage (160 allelic mismatches) and clonal group (25 allelic mismatches). The last six bins represent high-resolution levels of relatedness useful for epidemiological surveillance, with thresholds of 15, 8, 4, 2, 1 and 0 mismatches, respectively.

The LIN codes for the entire PGL dataset were assigned as described previously [50]. Briefly, a minimum spanning tree using Prim’s algorithm was created to define the order in which novel profiles are encoded, and based on this order, LIN codes were created for each distinct cgST.

When any new genome is added to the PGL, the genome is scanned for the 1222 core genes. Alleles are automatically assigned if hits with ≥97 % sequence identity and 100% sequence alignment length to exemplar alleles are found. Exemplar alleles are defined using find_exemplars.pl for each gene, ensuring all known alleles are within 3% sequence identity to an exemplar allele of the same length. Any alleles not automatically assigned are assessed by a human curator. A cgST is assigned to each genome with 25 or fewer missing core gene alleles. Subsequently, a LIN code for each cgMLST profile is assigned by matching it against all existing defined LIN-encoded cgSTs to identify its closest neighbour. If a match is found (i.e. same cgMLST profile), the same LIN code of the matching profile is assigned. Otherwise, a novel LIN code is generated as previously described [50]. Users submit their genomes and metadata to the PubMLST system for curation and assignment of cgMLST alleles, cgSTs and LIN codes (https://pubmlst.org/organisms/streptococcus-pneumoniae/submissions).

### Identification of population-wide variation in allelic mismatches

Pairwise allelic mismatch dissimilarities and the Silhouette index (*S*_t_) were assessed using MSTclust (v0.21b), using a subset of 5000 PGL genomes plus 50 *Streptococcus pseudopneumoniae* genomes (which provided detailed analyses in a reasonable processing time) and default parameters [27]. The *S*_t_, a measure of cluster cohesiveness, was calculated for the full range of pairwise allelic mismatches among the core genes. These analyses were repeated and validated on the entire dataset of 30 976 genomes, except for the Mandrake analysis, which was not performed on the full dataset due to the computationally intensive and time-consuming nature of extracting, aligning and analysing such a large number of sequences.

### Core genome alignment and phylogenetic analyses

A subset of 1900 genomes from the PGL was selected for phylogenetic analyses and comprised 1870 pneumococcal genomes chosen at random and included the 20 most prevalent STs plus 9 genomes of ST344 and 21 genomes of ST448. Fifty *S. pseudopneumoniae* genomes were included as an outgroup, chosen at random from a curated set of 77 *S*. *pseudopneumoniae* genomes from the rMLST database. The core gene sequences were retrieved by allele number from the sequence definitions database in PubMLST for each of the 1222 genes, aligned using MAFFT (v7.508; missing alleles were treated as gaps), and concatenated (total length 1.16 Mb) using a custom script [51,52]. A phylogenetic tree was created with FastTree (v2.1.11), using the generalized time-reversible model of nucleotide evolution and a single rate for each site (the ‘CAT’ approximation) [53]. This tree was reconstructed to account for recombination using ClonalFrameML [54]. This process was repeated for all lineage-specific phylogenetic analyses. The resulting phylogenetic trees were visualized using ggtree (v3.4.2), and cophenetic distance was calculated using the cophenetic function in the R stats package (v4.2.2) [55]. A minimum spanning tree was constructed using GrapeTree based on the 1222 cgMLST allelic profiles of the entire 30 976 PGL genomes [56].

### Comparison of clustering methods

CCs based on the seven-locus MLST scheme were assigned for all PGL isolates using the global optimal eBURST algorithm implemented in PHYLOViZ v2.0.0. CCs were defined at the single-locus variant level and named after the predicted founder sequence type(s) [57]. GPSCs were assigned using PopPunk (v2.6.5) and the GPS reference database (v8) [11,58]. Mandrake (v1.2.2) was used to cluster a concatenated alignment of the 1222 cgMLST genes from the randomly selected subset of 5000 PGL genomes stated above, using default parameters [12]. The adjusted Rand index (*R_t_*) was calculated for each classification level and each pre-existing metric (e.g. GPSC and CC) using the adjustedRandIndex function implemented in the mclust R package (v6.0.0) [59].

## Results

### A diverse global dataset of 30 976 published pneumococcal genomes

**Fig. 1.**
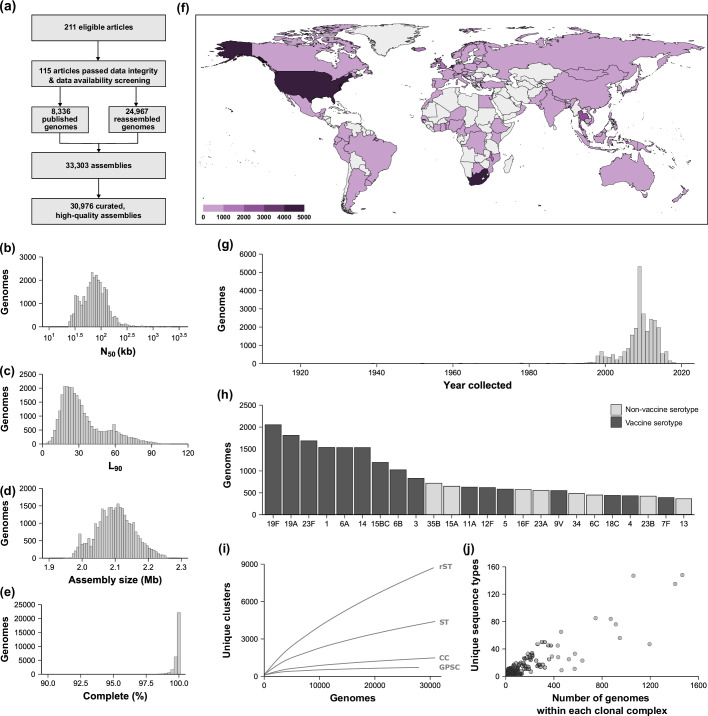
Summary of the creation and contents of the PGL. (**a**) Schematic overview of the creation of the PGL, containing 30 976 genome assemblies. (**b–d**) Assembly statistics for all genomes. (**e**) Number of complete and single-copy Benchmarking Universal Single-Copy Orthologs for all genomes. (**f**) Worldwide sampling density of the PGL, coloured by the number of genomes from each country. (**g**) Years of collection of the PGL pneumococci. (**h**) The 24 most prevalent serotypes in the PGL, marked to indicate serotypes included in any licensed pneumococcal vaccine (1, 2, 3, 4, 5, 6A, 6B, 7F, 8, 9N, 9V, 10A, 11A,12F, 14, 15BC, 17F, 18C, 19A, 19F, 20, 22F, 23F and 33F). (**i**) Rarefaction curves depicting the number of unique clusters observed for each of the four different taxonomic groups, plotted from random subsets of each sample size in triplicate. Ribosomal sequence type (rST), sequence type (ST; seven-locus multilocus sequence type), clonal complex (CC) and Global Pneumococcal Sequence Cluster (GPSC). (**j**) Number of unique sequence types within each clonal complex.

A total of 211 articles published in 78 journals between 2000 and 2020 that contained pneumococcal whole genome sequence data were identified (Figs 1a and S1, available in the online Supplementary Material). Just over half (*n* = 115, 54.5 %) of the publications provided access to all analysed genome data, including reference genomes and contextual isolates. The remaining articles either did not provide the entire genome dataset reported in the publication or there were data integrity issues. This included six publications that contained suppressed genomes, with no published corrections or clarifications regarding the reason for suppression or potential impact on published analyses. Some data issues were resolved by further investigation and/or contacting the corresponding authors for more information (Data S1).

Overall, the PGL was created with 33 303 genomes from 129 publications. Short-read data were downloaded and assembled for 24 967 genomes, and 8336 assembled genomes were downloaded from public repositories. In total, 30 976 (93.0 %) genomes passed all quality control metrics (Data S2 and S3). The genomes were generally highly contiguous (median *N*_50_ = 74.1 Kb; median *L*_90_ = 28 Kb; Fig. 1b, c) and of the expected size for a pneumococcal genome (median = 2.10 Mb, range = 1.95–2.26 Mb; Fig. 1d). Genomes typically resulted in a full representation of the Lactobacillales BUSCOs (median complete and single-copy BUSCOs = 100 %; Fig. 1e and Data S3; [46]). The remaining 2165 genomes (6.5 %) failed one or more quality checks, of which 67 showed evidence of contamination and 10 were not a pneumococcal genome (Data S3). They were excluded from further analyses and filtered out of the PGL.

Pneumococci included in the PGL were recovered from 82 countries across six continents, and more than half of the collection was from South Africa (*n* = 4887), the United States (*n* = 4270), the Netherlands (*n* = 3509), the Gambia (*n* = 2859) and Thailand (*n* = 2305; Fig. 1f; Data S4). Provenance data such as carriage/disease status and specimen source were available for 89.4 and 86.1 % of pneumococci, respectively. Overall, 42.6% of pneumococci were nasopharyngeal carriage samples. Pneumococci were recovered between 1916 and 2018, and 58.0% were recovered between 2009 and 2018 (Fig. 1g). Ninety-six serotypes were represented in the PGL, including 24 serotypes that are included in any licensed pneumococcal vaccine (Fig. 1h).

There were 8725 ribosomal sequence types (rSTs) and 4400 sequence types (STs, seven-locus MLST scheme) that clustered into 1482 CCs (Data S5 and S6) represented in the PGL. Variable-length-k-mer clustering identified 726 GPSCs (Data S5). Rarefaction analyses showed that the PGL effectively encompassed the known genetic diversity of pneumococcal population clusters as defined by CCs and GPSCs (Fig. 1i) but had a more limited representation of the entire known genotyping diversity as defined by pneumococcal STs and rSTs (>18 000 and >16 000, respectively, https://pubmlst.org/; Data S5). There was a positive association between the number of genomes within each CC and unique STs (Fig. 1j).

### Defining a pneumococcal core genome multilocus sequence typing scheme

Complete reference pneumococcal genomes (*n* = 42) were used to define a provisional set of 1385 non-redundant core genes (coding sequences with start and stop codons) that were present in at least 95% of the 42 complete genomes (Data S7). A subset of 7547 PGL genomes was then used to assess the allele assignments for each of the 1385 genes. Core genes with high numbers of disrupted or missing alleles were excluded (*n* = 163). This resulted in a final cgMLST v1.0 scheme of 1222 genes, which was consistent in size with previous estimates of the pneumococcal core genome (range = 912–1666 genes; [9,10, 60,62]).

The 1222 core genes were evenly distributed across the pneumococcal genome and had a diverse range of functions (Fig. S2 and Data S8). Per-locus analysis using the pairwise homoplasy index found evidence of intragenic recombination in 36.2% of these core genes (*n* = 442*; P* < 4.09×10^−5^ after Bonferroni correction; Fig. S3 and Data S9).

In total, 635 912 unique core gene alleles (range = 44–2776 alleles per gene) were assigned across the entire PGL (as of 30 April 2024; Data S10). 97.3 % of the PGL genomes were missing fewer than 25 allele assignments across all 1222 genes (Data S11). The number of core gene alleles assigned per genome did not vary substantially between the majority of CCs, suggesting minimal phylogenetic bias resulting from missing data (Figs S4 and S5). A cgST was assigned to each pneumococcus that had 25 or fewer missing core gene alleles (*n* = 30 141 genomes), which resulted in 29 647 unique cgSTs (Data S5).

For comparison, 50 genomes of *S. pseudopneumoniae*, a closely related species, from the rMLST database were screened for the presence of the 1222 pneumococcal core genes. Between 809 and 923 (median 884.5; 72.3 %) of the 1222 pneumococcal core genes were also identified among the *S. pseudopneumoniae* genomes, and alleles were assigned to those genes (Data S12); however, since all *S. pseudopneumoniae* genomes had more than 25 missing alleles, none were assigned a cgST, ensuring the scheme’s specificity for pneumococci.

### Defining the structure of pneumococcal populations using the cgMLST scheme

**Fig. 2.**
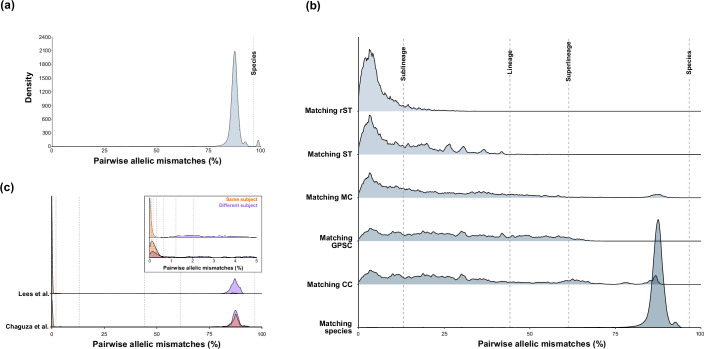
Distribution of pairwise cgMLST allelic differences across globally dispersed pneumococcal populations. (**a**) Pairwise cgMLST allelic differences between 5050 randomly selected PGL (*N* = 5000) and *S. pseudopneumoniae* (*N* = 50) genomes. *X*-axis values are plotted as a percentage of all 1222 cgMLST loci. (**b**) Distribution of pairwise cgMLST allelic differences between genomes belonging to the same taxonomic group: ribosomal sequence type (rST), sequence type (ST; seven-locus multilocus sequence type), Mandrake cluster (MC), Global Pneumococcal Sequence Cluster (GPSC), clonal complex (CC), and bacterial species. (**c**). Distribution of pairwise cgMLST allelic differences between pneumococci isolated from the same individual either concurrently (blood and cerebrospinal fluid samples) or longitudinally (1–52 weeks post-birth), as published by Lees *et al.* and Chaguza *et al.*, respectively [64,65].

A set of 5000 PGL genomes was chosen at random and used to set population structure boundaries. These boundaries were then validated using the entire PGL dataset. Pairwise allelic mismatches among the 1222 core genes formed a discontinuous distribution with three major peaks (Figs 2a and S6). The peak centred at 98.8% mismatches exclusively represented pairwise relationships between pneumococci and *S. pseudopneumoniae*. A high-level species classification boundary of 96.6% allelic mismatches among 1180 core genes was used to separate pneumococci from *S. pseudopneumoniae*. The peak centred at 93.0% mismatches predominantly represented comparisons between nontypable pneumococci of either CC344 or CC448 and other PGL pneumococci. CC344 and CC448 were previously implicated in conjunctivitis outbreaks and demonstrated a phylogenetic cluster distinct from other pneumococci [63,64]. The peak centred at 87.9% mismatches represented a diverse set of pneumococci sampled from different countries in different years.

There was no clear peak structure among pneumococci with fewer than 80 % pairwise mismatches (Figs 2a and S6). To define lineage boundaries within the data, the distributions of allelic mismatches within pneumococcal groups that were defined by ribosomal MLST, seven-locus MLST, Mandrake clusters, GPSCs and CCs were compared (Fig. 2b). In all cases, the distributions were positively skewed, and the majority of pairwise relationships were found below 50% mismatches.

Just over 98% of pneumococci within the same GPSC or Mandrake cluster, and 85 % of pneumococci within the same CC, had 61.4% (*n* = 750 genes) or fewer core gene allelic mismatches; thus, a ‘superlineage’ boundary was defined at 61.4% mismatches. Core gene allelic mismatches among pneumococci with the same ST ranged from 0.0 to 38.2% but were predominantly low (median = 6.6 % core gene allelic mismatches); therefore, a ‘lineage’ boundary (44.2% allelic mismatches or 540 genes) was defined to encompass all matching STs, which was among the highest *S*_t_ values.

rSTs catalogue sequence variation at ribosomal protein genes, which are conserved, robust to recombination effects, and typically used to differentiate bacterial species [18,65]. The PGL data demonstrated that pneumococci with matching rSTs also shared the majority of core gene alleles (97.9 % of pneumococci with matching rSTs had fewer than 13.1% (*n* = 160 genes) core gene allelic mismatches). A similar observation was made among STs (69.3% of pneumococci of the same ST had fewer than 13.1% core gene allelic mismatches); therefore, we defined a ‘sublineage’ boundary at 13.1% core gene mismatches.

Finally, to differentiate very closely related pneumococci more precisely, additional boundaries at 2.1%, 1.2%, 0.7%, 0.3%, 0.16% and 0.1 % (corresponding to 25, 15, 8, 4, 2 and 1 gene(s), respectively) were used. The 2.1% boundary defined ‘clonal group’ and the remaining boundaries defined ‘clonal subgroups’. Finally, a zero-mismatch boundary grouped cgMLST profiles that differed only by missing data. To validate these fine-scale boundaries, cgMLST allelic variations between pneumococci recovered from the same individuals either sampled concurrently (blood and cerebrospinal fluid; [66]) or longitudinally (1–52 weeks from birth; [67]) were compared (Fig. 2c). Pneumococci recovered from the same study subjects formed clear peaks at <1% core gene allelic mismatches (Fig. 2c).

### Multilevel clustering of PGL genomes

There were 29 647 unique cgSTs in the entire PGL. Multilevel single-linkage clustering was performed using one representative of each unique cgST, which iteratively clustered cgMLST profiles based on pairwise allelic mismatches, and cgST profiles below each mismatch threshold were assigned to the same cgST cluster. Clustering was performed sequentially, with the input order determined by the tip order of cgSTs in a minimum spanning tree that was constructed based on allelic profile similarity.

**Fig. 3.**
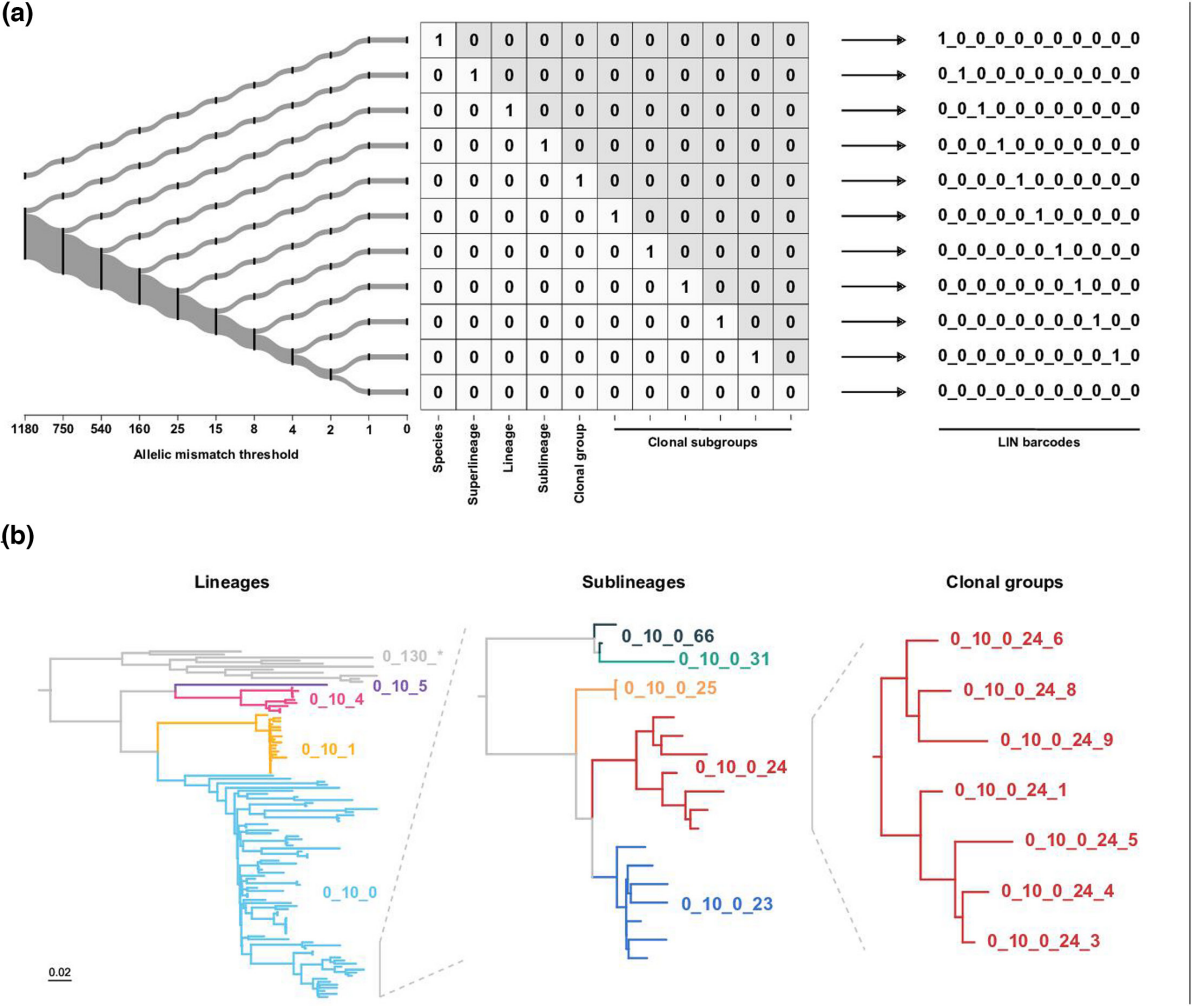
Schematic representation of the cgMLST LIN barcoding approach. (**a**) Overview of LIN barcode construction for each taxonomic level. A simplified phylogeny (left) depicts the allelic mismatch values used to define each single-linkage clustering threshold. The leftmost point of numerical dissimilarity in the barcode indicates the threshold at which genomes no longer cluster together. (**b**) A demonstration of phylogenetic relationships using superlineage 0_10 as an example and superlineage 0_130 as an outgroup. The relationships among lineages (left), sublineages (middle) and clonal groups (right) and the corresponding LIN barcodes are shown. (Note that for illustrative purposes, only a subset of all lineages is shown here.).

During cgST clustering, a cgMLST-based life identification number (cgLIN) code was also assigned to each cgST. This multi-positional barcode has 11 numbers that reflect the cgST cluster assignment at its respective threshold at the boundaries defined above, i.e. species, superlineage, lineage, sublineage, clonal group, clonal subgroups and the zero threshold. When any two barcodes were compared, the leftmost point of numerical dissimilarity reflected the threshold at which genomes no longer clustered together (Fig. 3a). Therefore, cgLIN barcodes reflected an approximation of the phylogenetic relationships between genomes, for example, superlineage 0_10 was composed of 4 lineages; lineage 0_10_0 was divided into 64 sublineages; and sublineage 0_10_0_24 subdivided into 10 distinct clonal groups (Fig. 3b).

In total, 27 749 unique cgLIN codes were defined for 30 141 PGL genomes (97.3% of all PGL genomes; Data S5). cgLIN codes defined 411 superlineages, 733 lineages, 2799 sublineages and 12 334 clonal groups within the PGL, and 72.3% of the lineages and 50.5% of the sublineages were represented by >1 genome (median, 4 genomes; range = 1–1467; Data S13 and S14). Over half of the PGL was represented by 15 superlineages and 23 lineages.

**Fig. 4.**
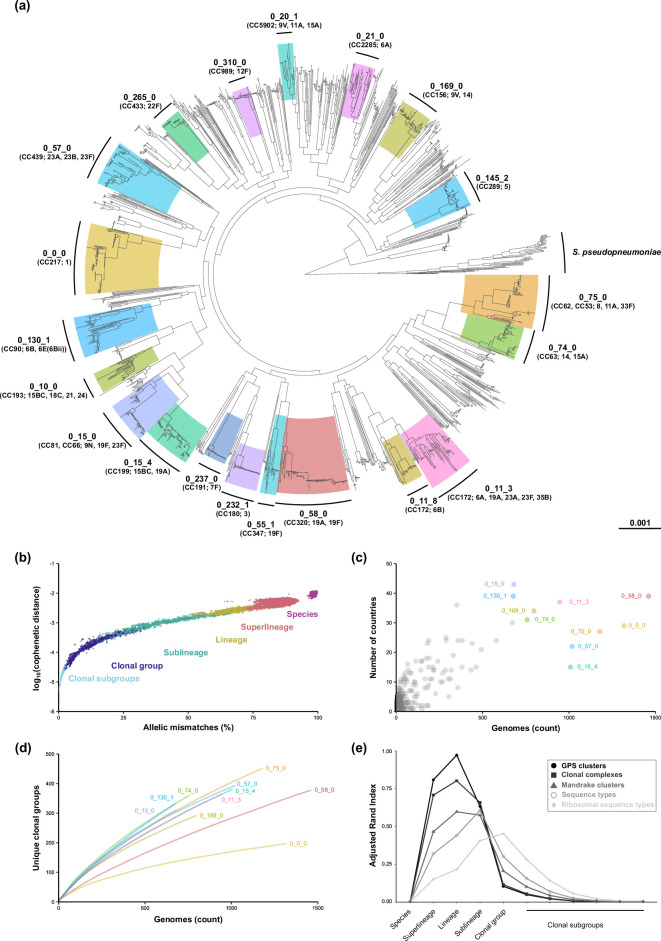
Global pneumococcal population structure and taxonomic classification. (**a**) Maximum-likelihood phylogeny of 1900 pneumococcal and 50 *S. pseudopneumoniae* genomes, based on a nucleotide alignment of 1222 cgMLST loci and rooted with *S. pseudopneumoniae*. The 20 most prevalent pneumococcal lineages are highlighted and annotated with CC, lineage LIN barcode (first three differentiating digits of the LIN barcode) and predominant serotype(s). (**b**) Comparison of similarity among genomes represented by the phylogeny in panel (a). Pairwise relationships were calculated for all 1900 genomes and randomly subset down to 50 000 pairs. Points are coloured by the closest inferred taxonomic relationship. (**c**) Geographical diversity of pneumococcal lineages. The 10 most prevalent lineages are highlighted. (**d**) Rarefaction curves depicting the number of unique clonal groups observed for each of the 10 most prevalent lineages, plotted from random subsets of each sample size in triplicate. (**e**) Similarity between cgMLST cluster classification levels defined in this publication and other measures of population structure. Concordance between clustering metrics was measured using the adjusted Rand index.

A subset of 1900 pneumococcal and 50 *S. pseudopneumoniae* genomes was chosen at random from the PGL and rMLST databases, respectively, to determine the phylogenetic congruence of the assigned lineages. A maximum-likelihood phylogeny was reconstructed using nucleotide sequence alignments of all 1222 cgMLST genes (Fig. 4a). The phylogeny identified 221 out of 733 lineages, and the 20 most prevalent lineages (47.4 % of PGL genomes) were predominantly monophyletic and formed discrete phylogroups. These findings were consistent with a minimum spanning tree visualization of clustering patterns using the cgST allelic profiles for the full dataset of 30 976 genomes (Fig. S7). Comparison of allelic dissimilarity and core genome divergence (represented by cophenetic distance of the phylogeny) demonstrated only limited overlap of classification levels (Fig. 4b).

Among lineages, 45.5% were comprised of pneumococci from more than one country, and 30.0% were from more than one continent (Data S4). Consistent with previous studies, individual lineages were normally associated with one or a small number of serotypes (mode number of serotypes per lineage, 1; range = 1–21 serotypes; Fig. S8 and Data S15). Rarefaction analysis suggested wide variability in substructure diversity within each lineage (Fig. 4d and Data S15). For example, lineage 0_0_0 (serotype 1, mainly CC217) demonstrated low clonal subgroup diversity despite extensive sampling (*n* = 1319 genomes), as compared to other lineages such as 0_15_0 (serotype 23F, mainly CC81 and CC66; *n =* 683) and 0_75_0 (serotype 11A, mainly CC53 and CC62; *n* = 1178). The concordance between cgLIN clustering and other approaches was calculated using the adjusted Rand index (ARI), a measure of similarity between clustering approaches. At the lineage level, cgLIN clustering was nearly identical to GPSCs (ARI = 0.97), highly concordant with CCs (ARI = 0.79) and Mandrake clusters (ARI = 0.6) (Fig. 4e).

## Discussion

Data sharing is essential for reproducibility and transparency in science. The open data movement has gained momentum, and many publishers, funders and organizations encourage or require authors to share data [68]. There are strong arguments for data sharing in the field of pathogen genomics, particularly in a public health context such as when managing disease outbreaks [3,69, 70]. The COVID-19 pandemic highlighted the benefits of rapid data sharing since publicly available SARS-CoV-2 genome data informed the development of vaccines, infection control strategies and diagnostic assays [71]. An open data culture allows datasets to be repurposed to advance our understanding of the epidemiology, evolution and biology of important human pathogens.

The PGL is a comprehensive, open access database of nearly 31 000 curated pneumococcal genomes from a broad range of countries, serotypes, genotypes, clinical manifestations and sampling years. The genomes and contextual data (provenance and phenotype) are easily accessible through the web-based PubMLST platform, which provides an extensive suite of third-party software to facilitate downstream analyses. The PGL aggregates existing genomes and helps to highlight underrepresented geographical regions that should be the focus of future genomic surveillance efforts.

Although the PGL contained genome data from 129 publications, many publications had data integrity issues that could not be resolved by the time of publication. (In the context of data and databases, ‘integrity’ refers to accurate, complete, valid and consistent data.) This is contrary to open data principles and policies and is a barrier to the reproduction of published analyses to assess the validity of research findings [72,76]. Importantly, it also precludes integration of these datasets into surveillance efforts, potentially leading to unnecessary duplication of efforts or distorting estimates of global coverage of pneumococcal genomic surveillance. More stringent checks of adherence to open data standards during the publication process are necessary and could be made easier by the standardization of data input formats. It also seems unlikely that these issues are unique to pneumococcal genomes and an assessment of published genome datasets for other microbial species is warranted.

Compared to the original seven-gene MLST, cgMLST provides a much higher resolution of pneumococcal population structure, comparable to that of single nucleotide variants or variable length k-mer comparisons. Moreover, by retaining the methodological approach and increasing the number of genes, cgMLST retains the advantages of MLST, namely consistency, standardized nomenclature, rapid allele assignment and the representation of alleles as numerical indices [16,25, 50, 77]. Furthermore, this cgMLST scheme is differentiated from other typing schemes by extensive manual curation of genes that were identified in a large validation set of genomes, which led to a large set of stable, reliably sequenced core genes and a robust genotyping scheme. Additionally, by applying this cgMLST scheme to the large PGL dataset, we have already created an extensive database of pneumococcal allelic variation, and this reduces the amount of curation required going forward as new genomes are added to the PGL.

Using a wide range of fixed clustering thresholds, the pneumococcal population was differentiated at varying phylogenetic and epidemiologically relevant scales, which provided greater resolution than typing schemes with single clustering levels. The added complexity of multilevel clustering was counterbalanced with an intuitive barcoding system and classification level, to provide a consistent description of each clustering level [50]. These analyses revealed many pneumococcal lineages, but since there were few obvious discontinuities in the percentage of pairwise allelic mismatches across the PGL, existing clustering metrics were used to guide the definition of LIN boundaries. The observed flat structure in pairwise allelic mismatches might be explained by geographical, serological or genotype-specific population substructures that are obscured by the large size of the PGL or might be due to the naturally high recombination rates of pneumococci that may have created a natural gradient of allelic similarity. The analyses of clonal groups and clonal subgroups aimed to differentiate very closely related pneumococci and those boundaries were validated with studies of multiple and longitudinal sampling. Finally, the observed concordance between methods that use different input sequence data allows investigators to choose MLST, or genomic analyses, and provides a degree of backwards compatibility with other clustering methods (e.g. CCs). The sequences at MLST loci can be determined either by PCR amplification and sequencing or by extracting the relevant sequences from a whole genome sequence, whereas a genome sequence is required for cgST/LIN codes, GPSCs and Mandrake clusters.

In conclusion, we have created a high-quality, open access genomic resource that is representative of pneumococcal global diversity and a novel pneumococcal cgMLST scheme and LIN barcoding system to define and evaluate genetic lineages, in a manner that reflects the complex structure of pneumococcal populations.

## supplementary material

10.1099/mgen.0.001280 Uncited Supplementary Material 1.

10.1099/mgen.0.001280 Uncited Supplementary Material 2.

## Abbreviations

ARI: adjusted Rand index
BIGSdb: Bacterial Isolate Genome Sequence Database
BUSCO: Benchmarking Universal Single-Copy Orthologs
CC: clonal complexes
cgLIN: cgMLST-based life identification number
cgMLST: core genome MLST
cgST: core genome sequence type
COVID-19: coronavirus disease 2019
ENA: European Nucleotide Archive
GC: guanine cytosine
GPSCs: Global Pneumococcal Sequence Clusters
INSDC: International Nucleotide Sequence Database Collaboration
IPD: invasive pneumococcal disease
LCTN: lowest common taxonomic node
LIN: life identification number
MC: Mandrake cluster
MLST: multilocus sequence typing
NCBI: National Center for Biotechnology Information
PGL: Pneumococcal Genome Library
RefSeq: NCBI Reference Sequence Database
rMLST: ribosomal MLST
rST: ribosomal sequence type
ST: sequence type
S_t_: Silhouette index
UI: uncertainty interval
