# Supplementary material for: Development of the Pneumococcal Genome Library, a core genome multilocus sequence typing scheme, and a taxonomic life identification number barcoding system to investigate and define pneumococcal population structure
Source: Microb Genom. 2024 Aug 13;10(8):001280. doi: 10.1099/mgen.0.001280 (PMC11321556; doi:10.1099/mgen.0.001280)
Supplement: Uncited Supplementary Material 1. [file mgen-10-01280-s001.pdf]

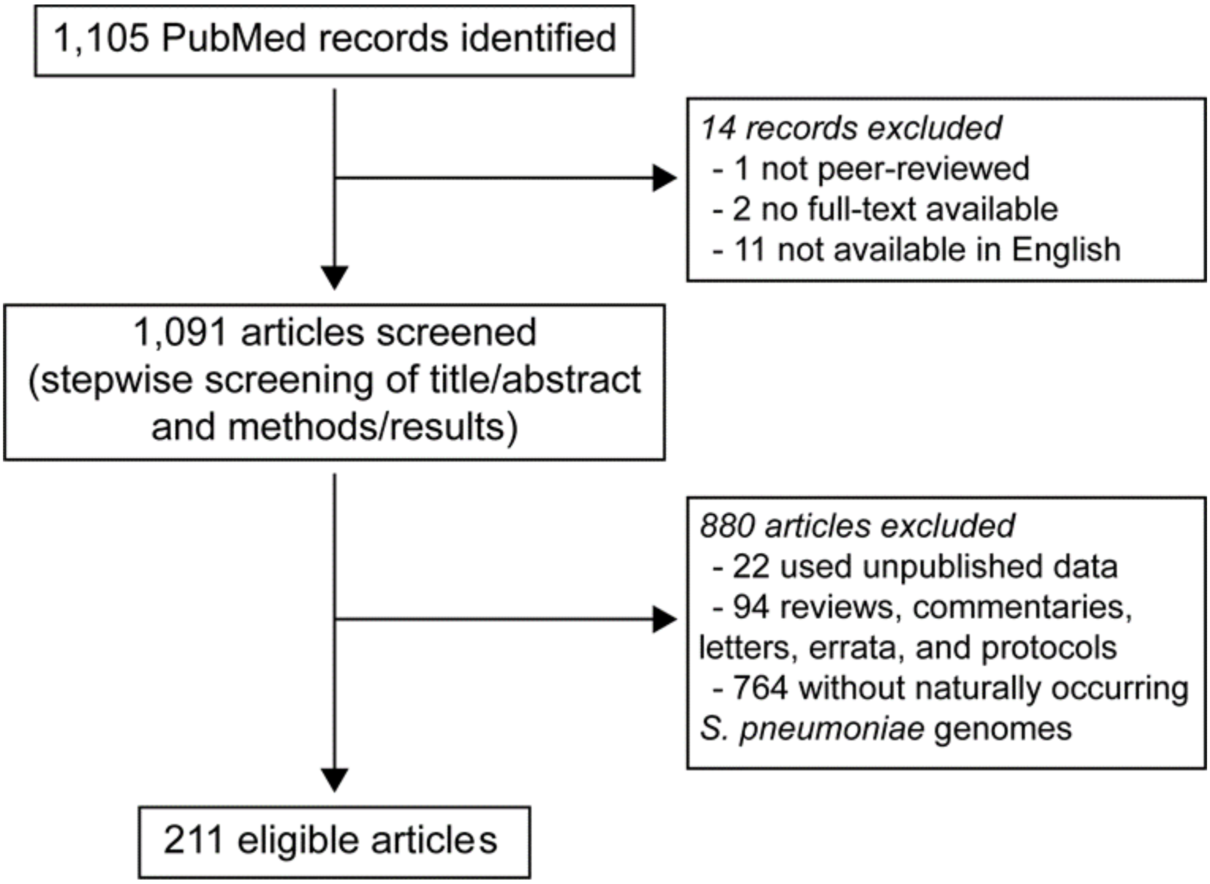

Supplementary Figure 1. Selection of publications for inclusion in the Pneumococcal Genome Library.

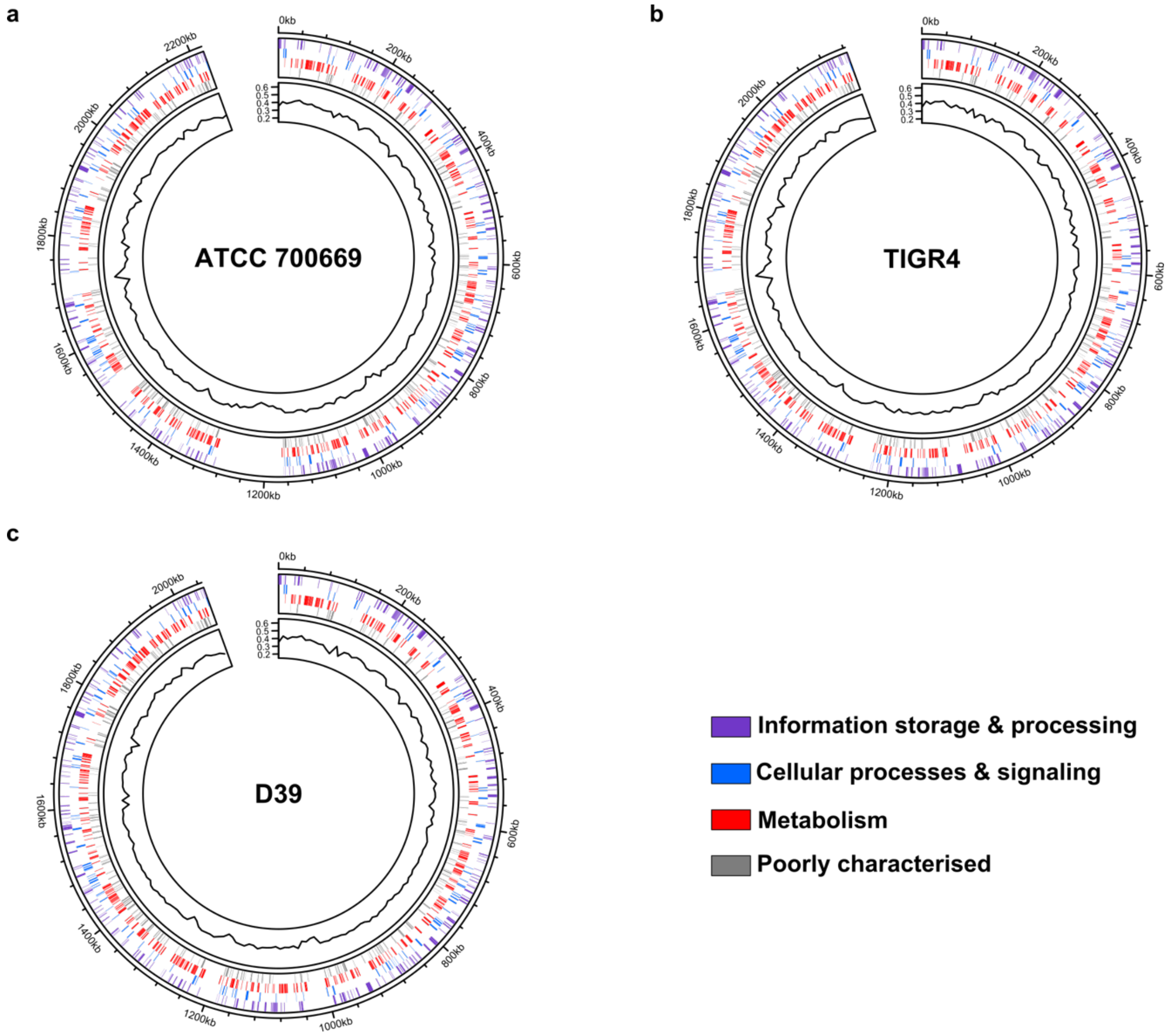

Supplementary Figure 2: Distribution of cgMLST loci across three closed (complete) pneumococcal reference genomes. NCBI RefSeq accession numbers for each reference genome are as follows: a) ATCC 700669, GCF_000026665.1; b) TIGR4, GCF_000006885.1; and c) D39, GCF_000014365.2. The outer ring in each plot represents cgMLST loci coloured by predicted gene function and Clusters of Orthologous Groups (COG) category for each gene cluster, as inferred by eggNOG-mapper. Loci without a predicted function were marked as ‘Poorly characterised’. The inner ring depicts the GC content, calculated as the median value of 1 kb windows along each complete genome.

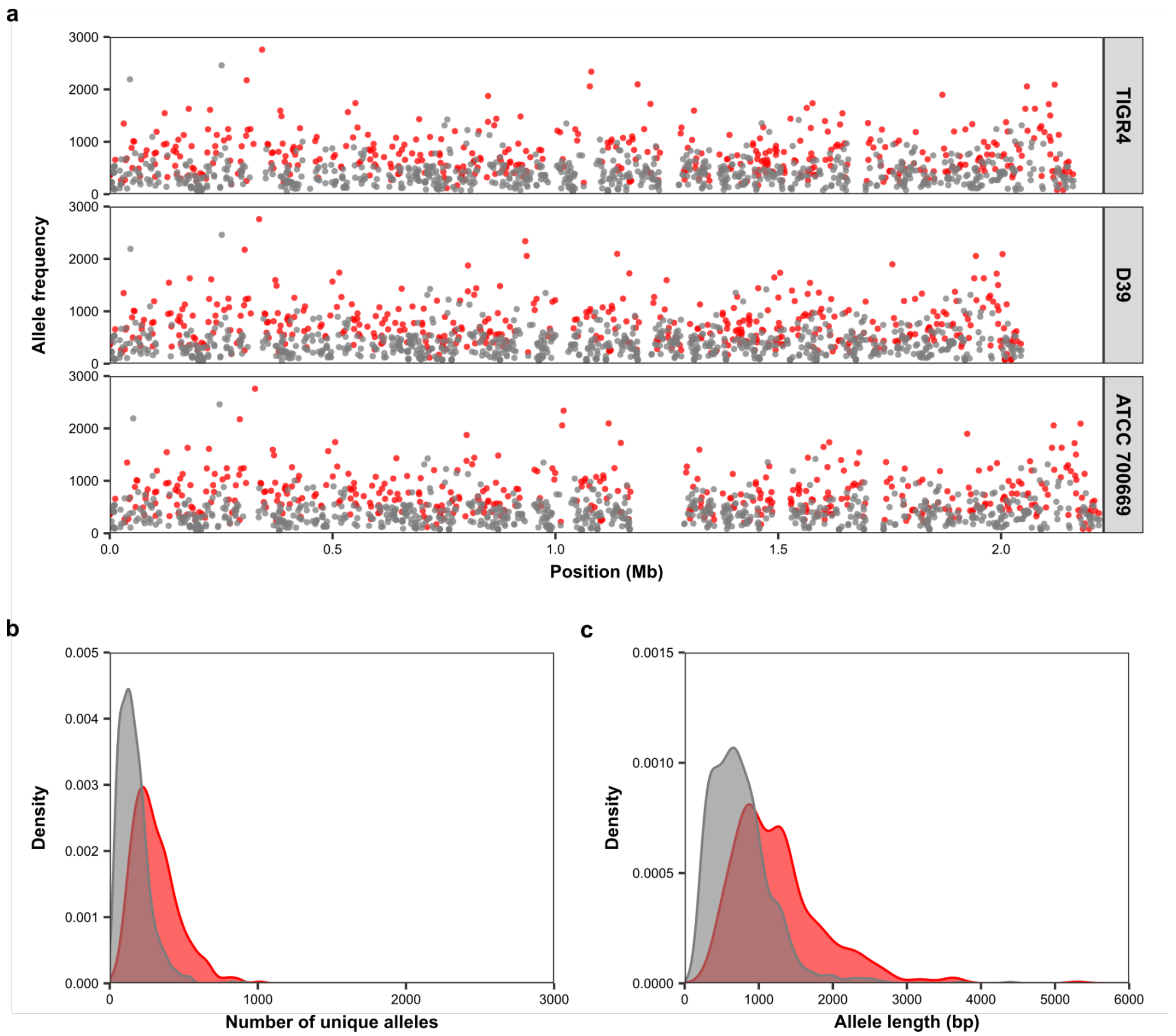

Supplementary Figure 3: Allele and recombination frequencies of the cgMLST core genes. Red points and peaks represent loci with evidence of significant intra-gene recombination, as estimated by the pairwise homoplasy index after Bonferroni correction ($p < 4.09 \times 10^{-5}$) compared to those loci without significant recombination (grey). a) Allele frequency at each cgMLST gene across three pneumococcal reference genomes. b) Distribution of total counts of unique alleles for each cgMLST genes (n = 1,222). c) Allele length distribution for each cgMLST gene (n = 1,222).

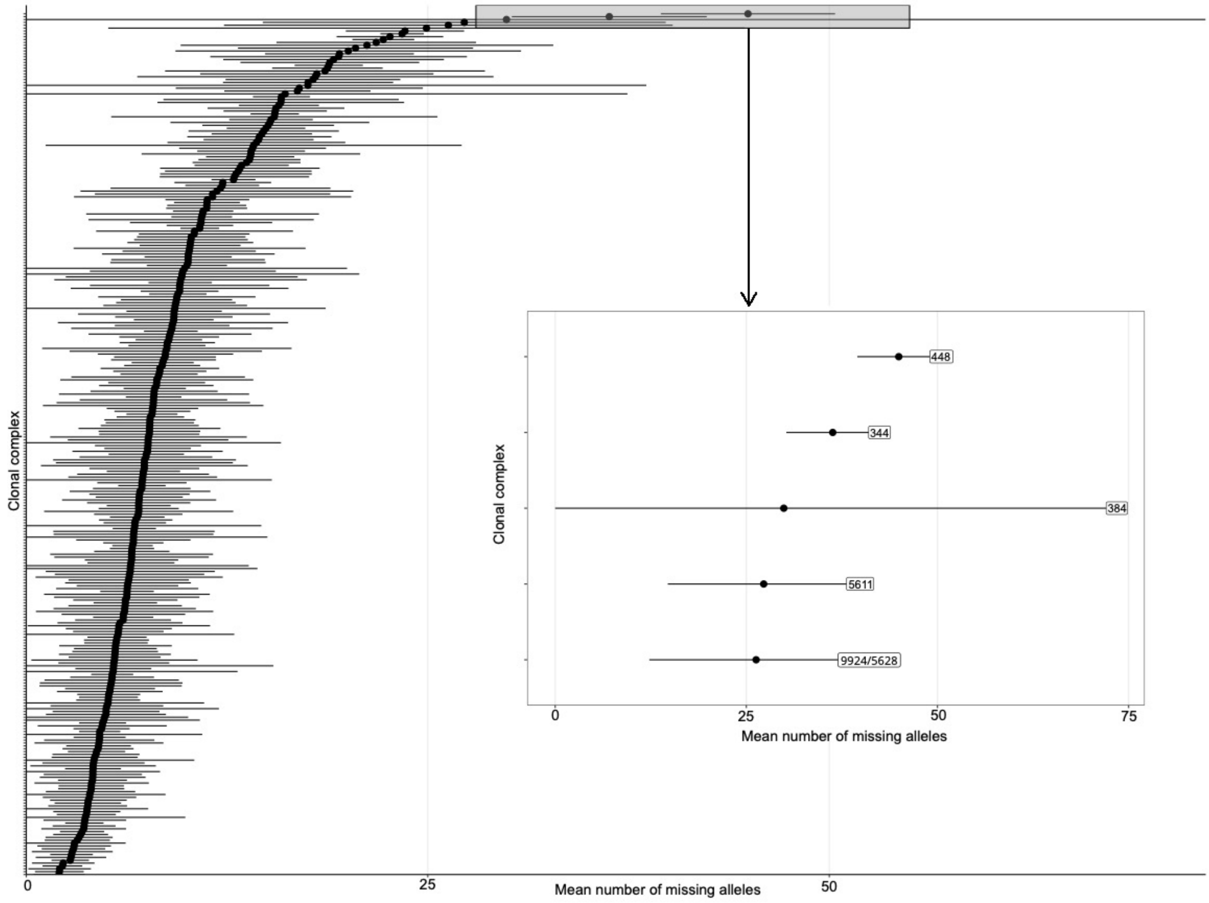

Supplementary Figure 4: Core gene allele assignments for each clonal complex. Black points represent the per-genome mean number of cgMLST genes without an allele assignment for each clonal complex, for all clonal complexes with >= 10 genomes. Horizontal lines represent the standard deviation of the mean. Clonal complexes with a mean >25 missing alleles are labelled.

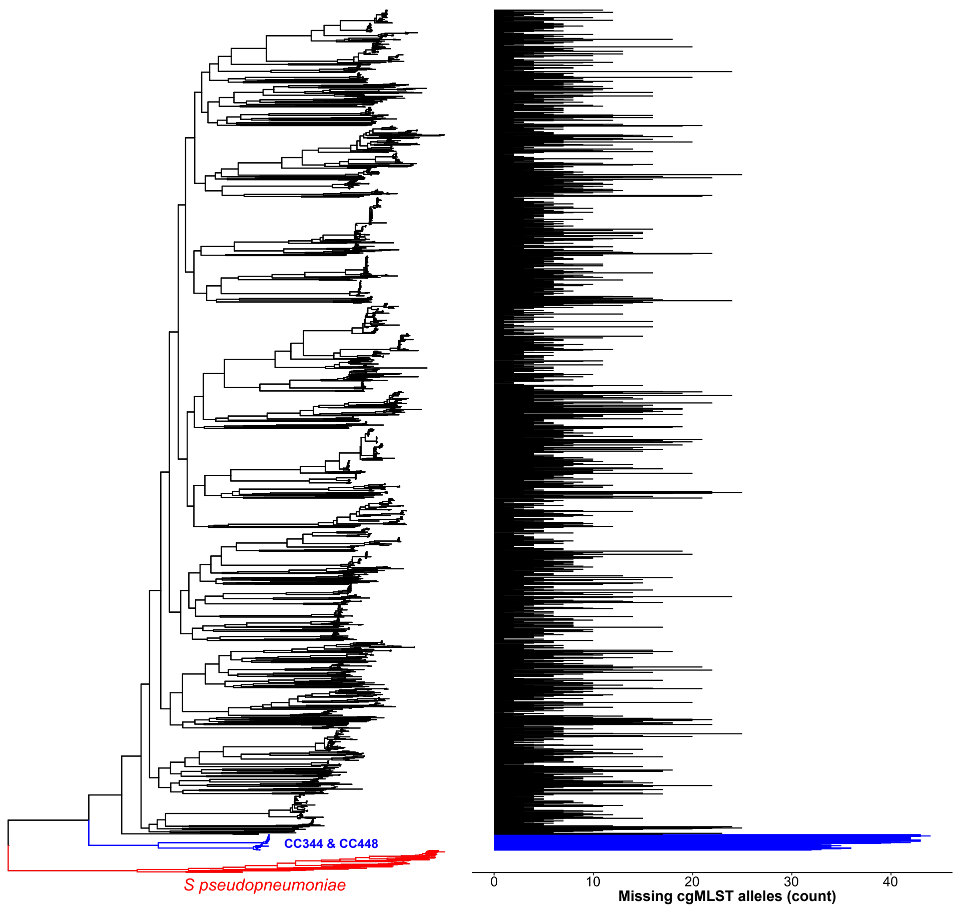

Supplementary Figure 5: Left: Maximum-likelihood phylogenetic tree representing data from 1,900 randomly selected PGL genomes plus 50 *S. pseudopneumoniae* genomes (red); representatives of CC344 and CC448 are highlighted in blue. The phylogenetic tree was constructed using a nucleotide alignment of 1,222 cgMLST loci and rooted with *S pseudopneumoniae*. Right: Per-genome rates of allele missingness (cgMLST genes with unassigned alleles), out of a total of 1,222 genes. Bars representing values of clonal complexes 344 & 448 are highlighted in blue.

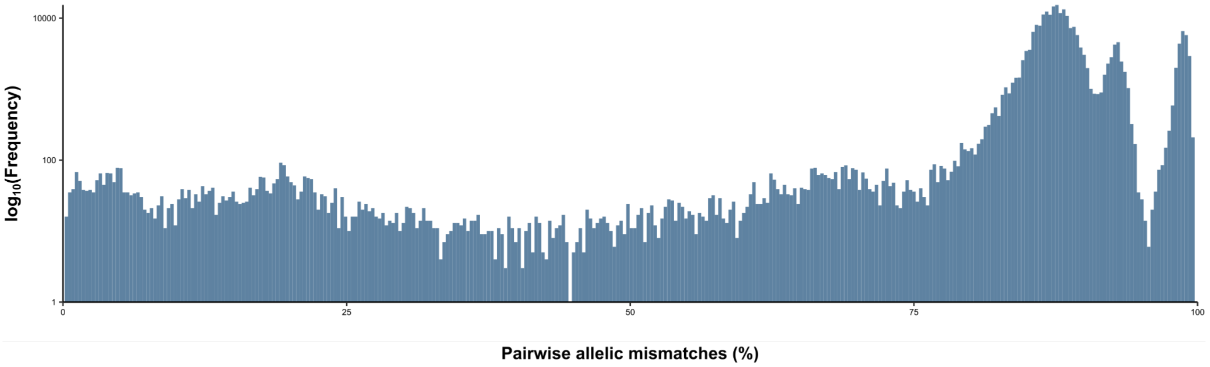

Supplementary Figure 6: Distribution of pairwise cgMLST allelic differences between 5,050 randomly selected PGL (N=5,000) and *S pseudopneumoniae* (N=50) genomes. Allelic mismatches on the x-axis are plotted as a percentage of all 1,222 cgMLST loci.

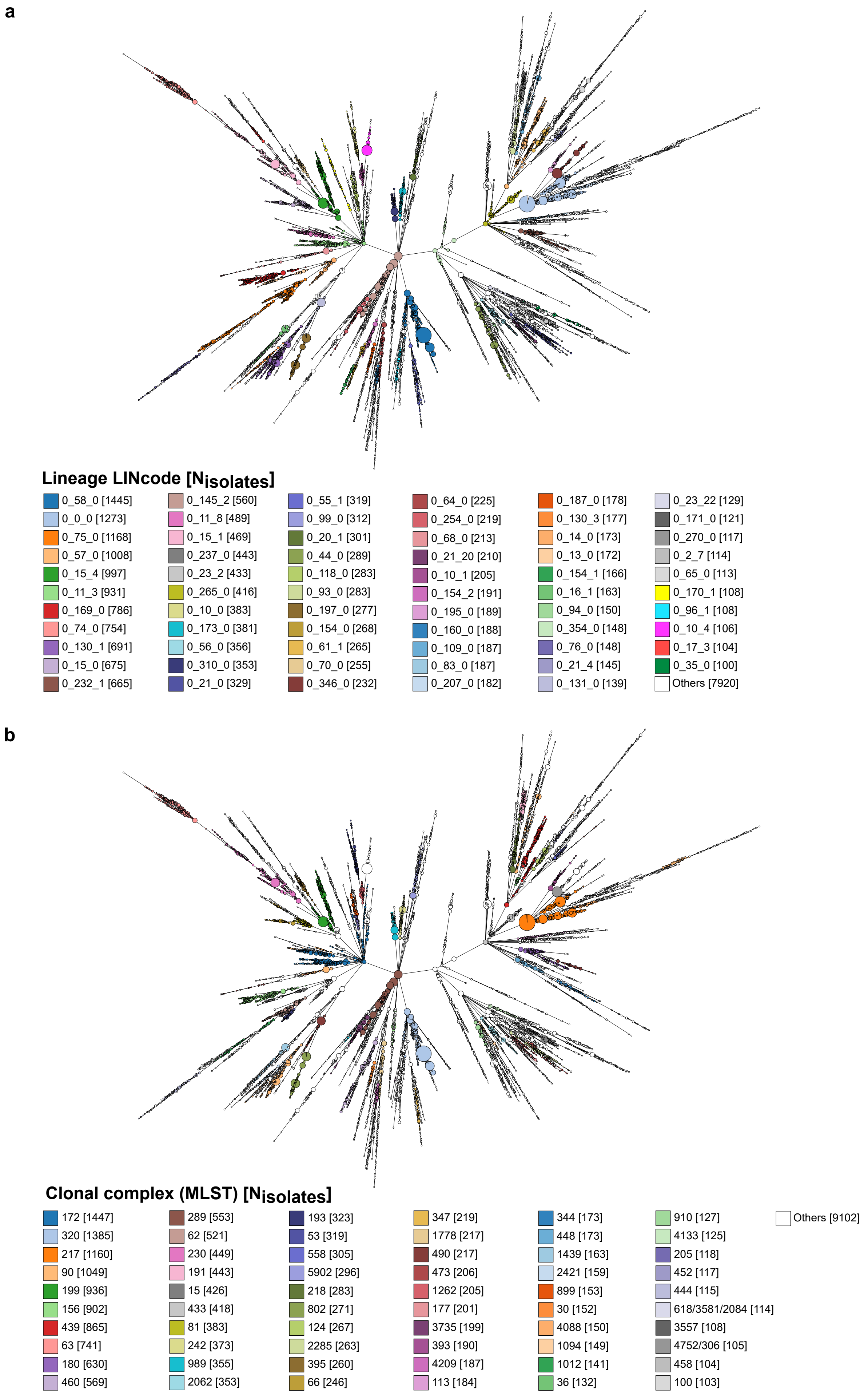
a
Lineage LINcode [Nisolates]
0_58_0 [1445]
0_0_0 [1273]
0_75_0 [1168]
0_57_0 [1008]
0_15_4 [997]
0_11_3 [931]
0_169_0 [786]
0_74_0 [754]
0_130_1 [691]
0_15_0 [675]
0_232_1 [665]
0_145_2 [560]
0_11_8 [489]
0_15_1 [469]
0_237_0 [443]
0_23_2 [433]
0_265_0 [416]
0_10_0 [383]
0_173_0 [381]
0_56_0 [356]
0_310_0 [353]
0_21_0 [329]
0_55_1 [319]
0_99_0 [312]
0_20_1 [301]
0_44_0 [289]
0_118_0 [283]
0_93_0 [283]
0_197_0 [277]
0_154_0 [268]
0_61_1 [265]
0_70_0 [255]
0_346_0 [232]
0_64_0 [225]
0_254_0 [219]
0_68_0 [213]
0_21_20 [210]
0_10_1 [205]
0_154_2 [191]
0_195_0 [189]
0_160_0 [188]
0_109_0 [187]
0_83_0 [187]
0_207_0 [182]
0_187_0 [178]
0_130_3 [177]
0_14_0 [173]
0_13_0 [172]
0_154_1 [166]
0_16_1 [163]
0_94_0 [150]
0_354_0 [148]
0_76_0 [148]
0_21_4 [145]
0_131_0 [139]
0_23_22 [129]
0_171_0 [121]
0_270_0 [117]
0_2_7 [114]
0_65_0 [113]
0_170_1 [108]
0_96_1 [108]
0_10_4 [106]
0_17_3 [104]
0_35_0 [100]
Others [7920]
b
Clonal complex (MLST) [Nisolates]
172 [1447]
320 [1385]
217 [1160]
90 [1049]
199 [936]
156 [902]
439 [865]
63 [741]
180 [630]
460 [569]
289 [553]
62 [521]
230 [449]
191 [443]
15 [426]
433 [418]
81 [383]
242 [373]
989 [355]
2062 [353]
193 [323]
53 [319]
558 [305]
5902 [296]
218 [283]
802 [271]
124 [267]
2285 [263]
395 [260]
66 [246]
347 [219]
1778 [217]
490 [217]
473 [206]
1262 [205]
177 [201]
3735 [199]
393 [190]
4209 [187]
113 [184]
344 [173]
448 [173]
1439 [163]
2421 [159]
899 [153]
30 [152]
4088 [150]
1094 [149]
1012 [141]
36 [132]
910 [127]
4133 [125]
205 [118]
452 [117]
444 [115]
618/3581/2084 [114]
3557 [108]
4752/306 [105]
458 [104]
100 [103]
Others [9102]

Supplementary Figure 7: Visualisation of clustering patterns among 30,976 genomes using the minimum spanning tree algorithm implemented in GrapeTree. Trees are annotated with the designated lineage as determined by LIN code in panel a, and with clonal complex in panel b.

a

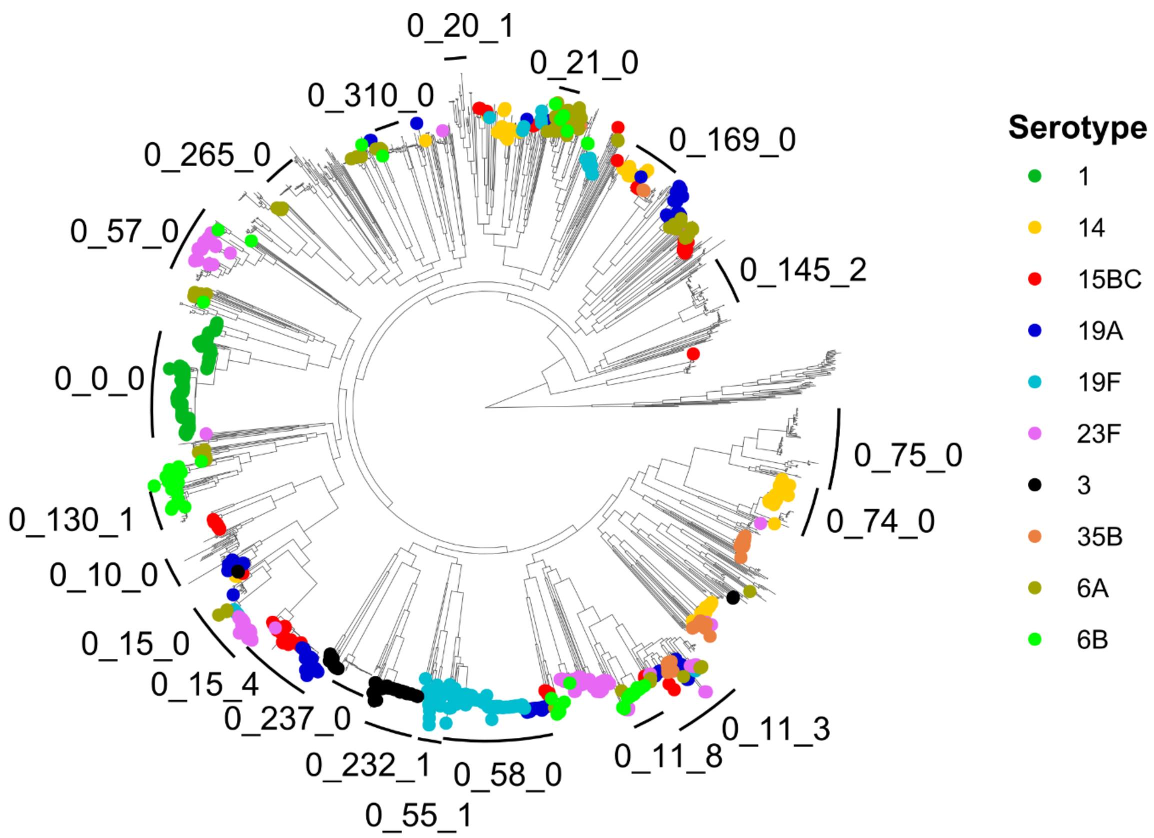
0_20_1
0_21_0
0_310_0
0_265_0
0_169_0
0_57_0
0_145_2
0_0_0
0_75_0
0_130_1
0_74_0
0_10_0
0_15_0
0_15_4
0_237_0
0_11_3
0_11_8
0_232_1
0_58_0
0_55_1
Serotype
1
14
15BC
19A
19F
23F
3
35B
6A
6B

b

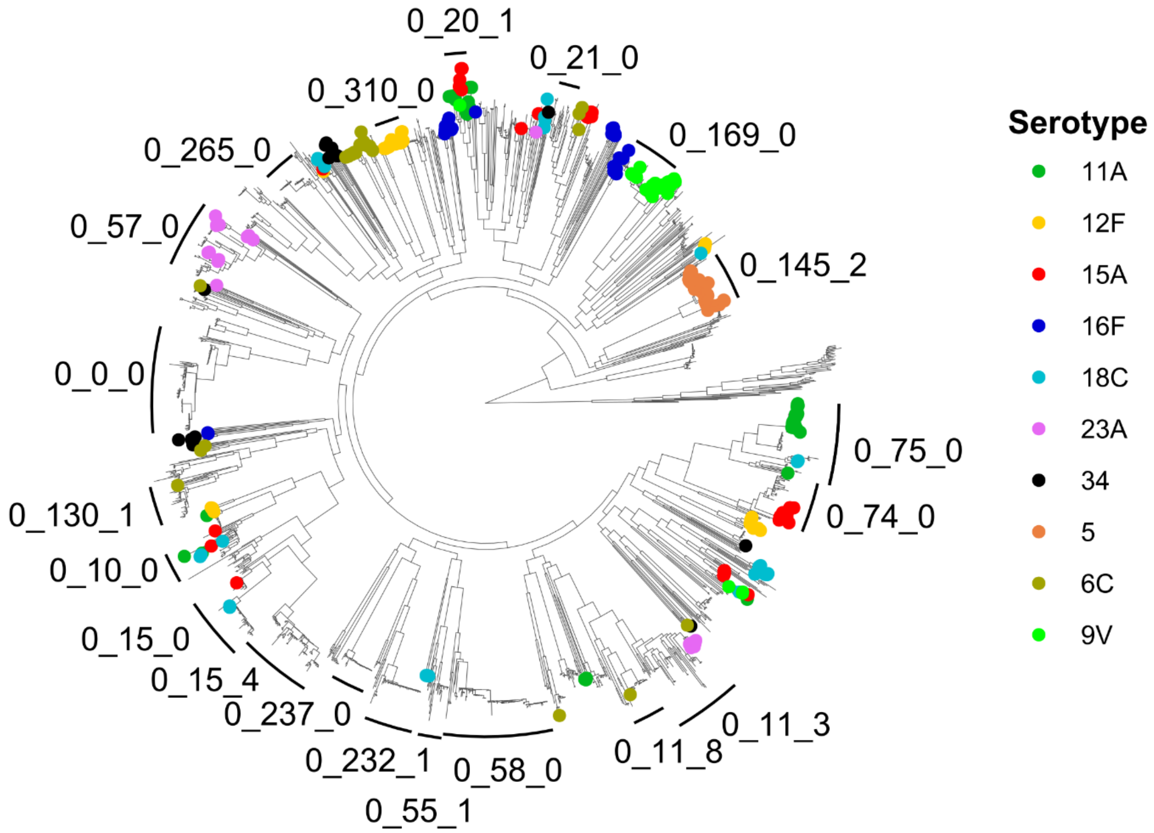
0_20_1
0_21_0
0_310_0
0_265_0
0_169_0
0_57_0
0_145_2
0_0_0
0_75_0
0_130_1
0_74_0
0_10_0
0_15_0
0_15_4
0_237_0
0_11_3
0_11_8
0_232_1
0_58_0
0_55_1
Serotype
11A
12F
15A
16F
18C
23A
34
5
6C
9V

Supplementary Figure 8: Maximum-likelihood phylogenetic tree representing data from 1,900 randomly selected PGL genomes plus 50 *S pseudopneumoniae* genomes. The phylogenetic tree was constructed using a nucleotide alignment of 1,222 cgMLST loci and rooted with *S pseudopneumoniae*. Genomes representing the 20 most prevalent serotypes are shown by coloured branch tips.
